# Crystal structure of (4-meth­oxy­phen­yl)[(4-meth­oxy­phen­yl)phospho­nato]dioxidophosphate(1−) 2-amino-6-benzyl-3-eth­oxy­carbon­yl-4,5,6,7-tetra­hydro­thieno[2,3-*c*]pyridin-6-ium

**DOI:** 10.1107/S2056989015022331

**Published:** 2015-11-28

**Authors:** Joel T. Mague, Shaaban K. Mohamed, Mehmet Akkurt, Sabry H. H. Younes, Essam K. Ahmed, Mustafa R. Albayati

**Affiliations:** a Department of Chemistry, Tulane University, New Orleans, LA 70118, USA; b Chemistry and Environmental Division, Manchester Metropolitan University, Manchester, M1 5GD, England; c Chemistry Department, Faculty of Science, Minia University, 61519 El-Minia, Egypt; d Department of Physics, Faculty of Sciences, Erciyes University, 38039 Kayseri, Turkey; e Chemistry Department, Faculty of Science, Sohag University, 82524 Sohag, Egypt; f Kirkuk University, College of Science, Department of Chemistry, Kirkuk, Iraq

**Keywords:** crystal structure, thieno­pyridines, Lawson reagent, mol­ecular salt, hydrogen bonding

## Abstract

The asymmetric unit of the title mol­ecular salt, C_17_H_21_N_2_O_2_S^+^·C_14_H_15_O_7_P_2_^−^, comprises two cations and two anions. Each cation features an intra­molecular N—H⋯O hydrogen bond, which closes an *S*(6) ring; in each case the hydro­pyridine ring adopts a half-chair conformation. In the anions, the dihedral angles between the aromatic rings are 64.1 (2) and 54.9 (2)°. In each case, the diphosphate groups are close to eclipsed [C—P⋯P—C pseudo-torsion angles = 11.6 (2) and −19.3 (2)°]. One of the meth­oxy groups in each anion is disordered over two orientations in a 0.539 (18):0.461 (18) ratio in one anion and 0.82 (2):0.18 (2) in the other. In the crystal, O—H⋯O and N—H⋯O hydrogen bonds link the components into [100] chains. Numerous C—H⋯O inter­actions cross-link the chains into a three-dimensional network.

## Related literature   

For the synthesis and biological applications of tetra­hydro­thieno­pyridines, see: Grunewald *et al.* (2008 ▸); Baker & White (2009 ▸); Boschelli *et al.* (2005 ▸). For the diverse biological activities of thieno­pyridines, see: Huber *et al.* (2009 ▸); Taniuchi *et al.* (2001 ▸); Bernardinoa & Pinheiroa (2006 ▸); Tumey *et al.* (2008 ▸); Attaby *et al.* (1999 ▸), Grunewald *et al.* (2008 ▸), Andersen *et al.* (2002 ▸). For a similar structure, see: Kingsley *et al.* (2001 ▸).
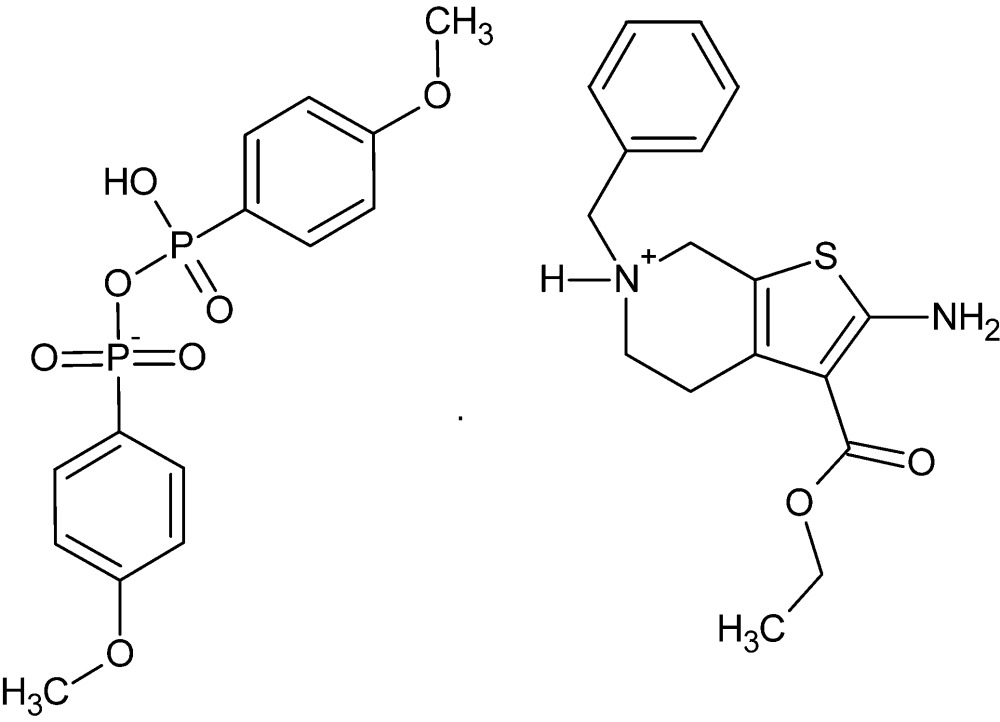


## Experimental   

### Crystal data  

C_17_H_21_N_2_O_2_S^+^·C_14_H_15_O_7_P_2_^−^
*M* *_r_* = 674.62
Triclinic, 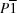

*a* = 12.443 (2) Å
*b* = 15.194 (3) Å
*c* = 17.856 (3) Å
α = 107.099 (2)°
β = 90.376 (2)°
γ = 96.899 (2)°
*V* = 3200.4 (10) Å^3^
*Z* = 4
Mo *K*α radiation
μ = 0.26 mm^−1^
*T* = 150 K
0.22 × 0.18 × 0.09 mm

### Data collection  

Bruker SMART APEX CCD diffractometer
Absorption correction: multi-scan (*SADABS*; Bruker, 2015 ▸) *T*_min_ = 0.72, *T*_max_ = 0.98
27878 measured reflections
13602 independent reflections
6997 reflections with *I* > 2σ(*I*)
*R*_int_ = 0.066

### Refinement  

*R*[*F* ^2^ > 2σ(*F* ^2^)] = 0.064
*wR*(*F* ^2^) = 0.183
*S* = 0.99
13602 reflections
829 parameters
41 restraints
H-atom parameters constrained
Δρ_max_ = 0.79 e Å^−3^
Δρ_min_ = −0.37 e Å^−3^

### 

Data collection: *APEX2* (Bruker, 2015 ▸); cell refinement: *SAINT* (Bruker, 2015 ▸); data reduction: *SAINT*; program(s) used to solve structure: *SHELXT* (Sheldrick, 2015*a* ▸); program(s) used to refine structure: *SHELXL2014/7* (Sheldrick, 2015*b* ▸); molecular graphics: *DIAMOND* (Brandenburg & Putz, 2012 ▸); software used to prepare material for publication: *SHELXTL* (Sheldrick, 2008 ▸).

## Supplementary Material

Crystal structure: contains datablock(s) global, I. DOI: 10.1107/S2056989015022331/hb7549sup1.cif

Structure factors: contains datablock(s) I. DOI: 10.1107/S2056989015022331/hb7549Isup2.hkl

**Fig. 1.**
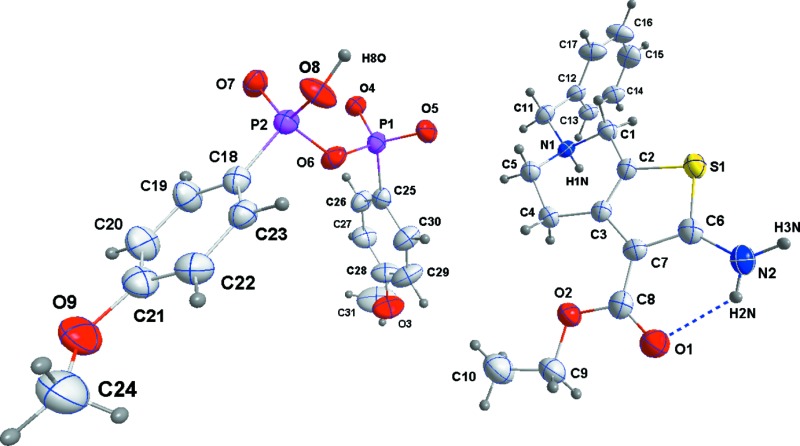
Title molecule 1 with labeling scheme and 50% probability ellipsoids. The intramolecular N—H···O hydrogen bond is shown by a dotted line.

. DOI: 10.1107/S2056989015022331/hb7549fig1.tif
Title mol­ecule 1 with labeling scheme and 50% probability ellipsoids. The intra­molecular N—H⋯O hydrogen bond is shown by a dotted line.

**Fig. 2.**
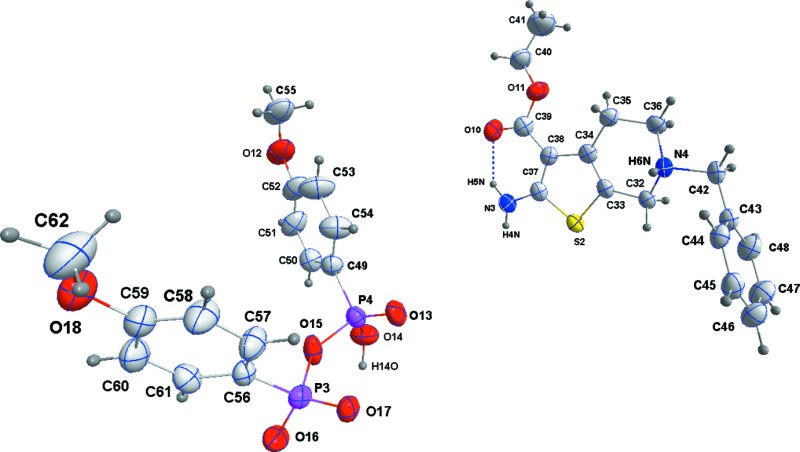
Title molecule 2 with labeling scheme and 50% probability ellipsoids. The intramolecular N—H···O hydrogen bond is shown by a dotted line.

. DOI: 10.1107/S2056989015022331/hb7549fig2.tif
Title mol­ecule 2 with labeling scheme and 50% probability ellipsoids. The intra­molecular N—H⋯O hydrogen bond is shown by a dotted line.

**Fig. 3.**
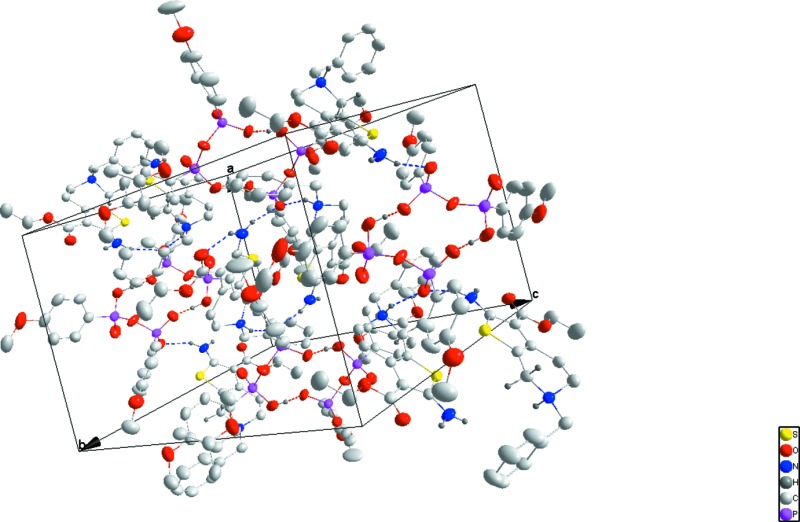
Packing projected onto (011). Intermolecular N—H···O and O—H···O hydrogen bonds are shown, respectively, by blue and red dotted lines.

. DOI: 10.1107/S2056989015022331/hb7549fig3.tif
Packing projected onto (011). Inter­molecular N—H⋯O and O—H⋯O hydrogen bonds are shown, respectively, by blue and red dotted lines.

CCDC reference: 1438194

Additional supporting information:  crystallographic information; 3D view; checkCIF report

## Figures and Tables

**Table 1:** Hydrogen-bond geometry (Å, °)

*D*—H⋯*A*	*D*—H	H⋯*A*	*D*⋯*A*	*D*—H⋯*A*
N1—H1*N*⋯O13	0.91	2.37	2.853 (4)	113
N1—H1*N*⋯O17	0.91	2.06	2.924 (4)	159
N2—H2*N*⋯O1	0.91	2.07	2.767 (4)	132
N2—H3*N*⋯O17^i^	0.91	2.06	2.910 (4)	155
N3—H4*N*⋯O4^ii^	0.91	2.07	2.946 (5)	162
N3—H5*N*⋯O10	0.91	2.01	2.763 (6)	139
N4—H6*N*⋯O4	0.91	2.00	2.832 (4)	151
N4—H6*N*⋯O7	0.91	2.51	3.001 (4)	114
O8—H8*O*⋯O5^iii^	0.98	1.46	2.434 (4)	175
O14—H14*O*⋯O16^iv^	0.98	1.42	2.374 (4)	164
C1—H1*A*⋯O7^iii^	0.99	2.59	3.432 (4)	143
C5—H5*A*⋯O13	0.99	2.51	2.999 (5)	110
C11—H11*A*⋯O13	0.99	2.58	3.144 (5)	116
C14—H14⋯O15^iv^	0.95	2.57	3.466 (5)	158
C20—H20⋯O18^v^	0.95	2.32	3.119 (6)	141
C27—H27⋯O10	0.95	2.60	3.469 (5)	153
C36—H36*B*⋯O7	0.99	2.54	3.146 (5)	119
C41—H41*A*⋯O3^vi^	0.98	2.40	3.09 (2)	127
C42—H42*B*⋯O7	0.99	2.39	3.073 (5)	125
C46—H46⋯O9^vii^	0.95	2.60	3.508 (6)	161

Symmetry codes: (i) 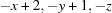; (ii) 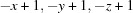; (iii) 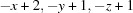; (iv) 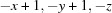; (v) 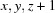; (vi) 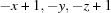; (vii) 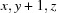.

## supplementary crystallographic information

### S1. Comment

Tetrahydrothienopyridine derivatives (THTPs) have received considerable attention due to their diverse biological activities such as anti-inflammatory and analgesic agents (Baker & White, 2009; Huber *et al.*, 2009; Taniuchi *et al.*, 2001; Andersen *et al.*, 2002; Boschelli *et al.*, 2005). Thieno-pyridine derivatives used in medicine as allosteric adenosine receptor and treatment of adenosine-sensitive cardiac arrhythmias (Bernardinoa & Pinheiroa, 2006; Tumey *et al.*, 2008; Attaby *et al.*, 1999, Grunewald *et al.*, 2008). In this context, we report in this study the synthesis and crystal structure of the title compound.

The asymmetric unit consists of two independent formula units in which the conformations of both the cations and the anions differ significantly between the two units as judged from overlays of the two independent cations and of the two independent anions. This is also evindent from Cremer-Pople puckerring analyses of the 6-membered heterocyclic rings in the cations. For the ring C1—C5,N1 the puckering parameters are Q = 0.487 (4) Å, θ = 50.0 (5)° and φ = 335.2 (6)° while for the ring C32—C36, N4 the values are Q = 0.510 (4) Å, θ = 128.5 (4)° and φ = 154.4 (6)°. The conformations of the cations are determined in part by intramolecular N2—H2n···O1 and N3—H5n···O10 hydrogen bonds (Table 1 and Figs. 1 and 2). Each anion forms a dimer with its centrosymmetrically related counterpart *via* pairwise O8—H8O···O5 and O14—H14O···O16 hydrogen bonds (Table 1 and Fig. 3) in which the H···O distances are notably short, although not quite as short as in the similar anion dimer found in [PdCl(3,5-Me_2_pz)_3_][PhP(O)(OH)OP(O)_2_Ph] (Kingsley, *et al.*, 2001).

### S2. Experimental

To a stirred solution of ethyl 2-amino-5-benzyl-4,5,6,7-tetrahydrothieno[3,2-*c*]pyridine-3-carboxylate (0.5 mmol, 158 mg) in acetonitrile (10 ml) was added 202.25 mg (0.5 mmol) of 2,4-bis(4-methoxyphenyl)-1,3,2,4-dithiadiphosphetane 2,4-disulfide (Lawesson's reagent, LR). The reaction was refluxed and monitored by TLC until completion (**ca** 6 h). The reaction was allowed to cool to room temperature, filtered off and washed with diethyl ether. The crude product was recrystallized from ethanol to afford pale yellow plates.

### S3. Refinement

H-atoms attached to carbon atoms were placed in calculated positions (C—H = 0.95 − 0.98 Å) while those attached to nitrogen atoms were placed in locations derived from a difference map and their coordinates adjusted to give N—H = 0.91%A. Those attached to oxygen atoms in the anion were initially refined to validate their presence and then fixed in the refined position to give O—H = 0.98. A l l were included as riding contributions with isotropic displacement parameters 1.2 − 1.5 times those of the attached atoms. The ethyl group in one cation and a methoxy group in one anion are disordered over two resolved sites. The components of each disorder were refined subject to restraints that their geometries be comparable.

### Crystal data

**Table:**

C_17_H_21_N_2_O_2_S^+^·C_14_H_15_O_7_P_2_^−^	*Z* = 4
*M**_r_* = 674.62	*F*(000) = 1416
Triclinic, *P*1	*D*_x_ = 1.400 Mg m^−^^3^
*a* = 12.443 (2) Å	Mo *K*α radiation, λ = 0.71073 Å
*b* = 15.194 (3) Å	Cell parameters from 4184 reflections
*c* = 17.856 (3) Å	θ = 2.2–23.1°
α = 107.099 (2)°	µ = 0.26 mm^−^^1^
β = 90.376 (2)°	*T* = 150 K
γ = 96.899 (2)°	Thick plate, pale yellow
*V* = 3200.4 (10) Å^3^	0.22 × 0.18 × 0.09 mm

### Data collection

**Table:**

Bruker SMART APEX CCD diffractometer	13602 independent reflections
Radiation source: fine-focus sealed tube	6997 reflections with *I* > 2σ(*I*)
Graphite monochromator	*R*_int_ = 0.066
Detector resolution: 8.3333 pixels mm^-1^	θ_max_ = 26.9°, θ_min_ = 1.2°
φ and ω scans	*h* = −15→15
Absorption correction: multi-scan (*SADABS*; Bruker, 2015)	*k* = −19→19
*T*_min_ = 0.72, *T*_max_ = 0.98	*l* = −22→22
27878 measured reflections	

### Refinement

**Table:**

Refinement on *F*^2^	Primary atom site location: structure-invariant direct methods
Least-squares matrix: full	Secondary atom site location: difference Fourier map
*R*[*F*^2^ > 2σ(*F*^2^)] = 0.064	Hydrogen site location: mixed
*wR*(*F*^2^) = 0.183	H-atom parameters constrained
*S* = 0.99	*w* = 1/[σ^2^(*F*_o_^2^) + (0.0581*P*)^2^] where *P* = (*F*_o_^2^ + 2*F*_c_^2^)/3
13602 reflections	(Δ/σ)_max_ = 0.001
829 parameters	Δρ_max_ = 0.79 e Å^−^^3^
41 restraints	Δρ_min_ = −0.37 e Å^−^^3^

### Special details

**Table:**

Experimental. The diffraction data were collected in three sets of 363 frames (0.5° width in ω) at φ = 0, 120 and 240°. A scan time of 40 sec/frame was used.
Geometry. All e.s.d.'s (except the e.s.d. in the dihedral angle between two l.s. planes) are estimated using the full covariance matrix. The cell e.s.d.'s are taken into account individually in the estimation of e.s.d.'s in distances, angles and torsion angles; correlations between e.s.d.'s in cell parameters are only used when they are defined by crystal symmetry. An approximate (isotropic) treatment of cell e.s.d.'s is used for estimating e.s.d.'s involving l.s. planes.
Refinement. Refinement of *F*^2^ against ALL reflections. The weighted *R*-factor *wR* and goodness of fit *S* are based on *F*^2^, conventional *R*-factors *R* are based on *F*, with *F* set to zero for negative *F*^2^. The threshold expression of *F*^2^ > σ(*F*^2^) is used only for calculating *R*-factors(gt) *etc*. and is not relevant to the choice of reflections for refinement. *R*-factors based on *F*^2^ are statistically about twice as large as those based on *F*, and *R*- factors based on ALL data will be even larger. H-atoms attached to carbon were placed in calculated positions (C—H = 0.95 − 0.98 Å) while those attached to nitrogen were placed in locations derived from a difference map and their coordinates adjusted to give N—H = 0.91%A. Those attached to oxygen in the anion were initially refined to validate their presence and then fixed in the refined position to give O—H = 0.98. A l l were included as riding contributions with isotropic displacement parameters 1.2 − 1.5 times those of the attached atoms. The ethyl group in one cation and a methoxy group in one anion are disordered over two resolved sites. The components of each disorder were refined subject to restraints that their geometries be comparable.

### Fractional atomic coordinates and isotropic or equivalent isotropic displacement parameters (Å^2^)

**Table:**

	*x*	*y*	*z*	*U*_iso_*/*U*_eq_	Occ. (<1)
S1	1.07706 (7)	0.50558 (7)	0.08459 (6)	0.0360 (3)	
O1	1.0854 (2)	0.19358 (19)	0.02015 (16)	0.0465 (7)	
O2	0.9462 (2)	0.19527 (18)	0.10000 (16)	0.0439 (7)	
N1	0.8015 (2)	0.51635 (19)	0.20406 (16)	0.0300 (7)	
H1N	0.7643	0.4944	0.1569	0.036*	
N2	1.1666 (2)	0.3609 (2)	−0.00291 (19)	0.0437 (9)	
H2N	1.1736	0.2997	−0.0127	0.052*	
H3N	1.2160	0.3993	−0.0197	0.052*	
C1	0.9083 (3)	0.5581 (3)	0.1841 (2)	0.0333 (9)	
H1A	0.9496	0.5964	0.2327	0.040*	
H1B	0.8969	0.5986	0.1511	0.040*	
C2	0.9707 (3)	0.4818 (3)	0.1409 (2)	0.0300 (8)	
C3	0.9516 (3)	0.3923 (3)	0.1375 (2)	0.0299 (8)	
C4	0.8610 (3)	0.3604 (3)	0.1828 (2)	0.0341 (9)	
H4A	0.8878	0.3199	0.2114	0.041*	
H4B	0.8014	0.3233	0.1458	0.041*	
C5	0.8184 (3)	0.4415 (3)	0.2406 (2)	0.0376 (9)	
H5A	0.7488	0.4196	0.2598	0.045*	
H5B	0.8704	0.4673	0.2862	0.045*	
C6	1.0929 (3)	0.3891 (3)	0.0511 (2)	0.0344 (9)	
C7	1.0226 (3)	0.3359 (3)	0.0857 (2)	0.0303 (8)	
C8	1.0233 (3)	0.2377 (3)	0.0653 (2)	0.0369 (9)	
C9	0.9420 (4)	0.0952 (3)	0.0803 (3)	0.0546 (12)	
H9A	1.0127	0.0783	0.0932	0.066*	
H9B	0.9244	0.0660	0.0235	0.066*	
C10	0.8560 (4)	0.0635 (3)	0.1274 (3)	0.0756 (16)	
H10A	0.8731	0.0948	0.1833	0.113*	
H10B	0.8521	−0.0038	0.1174	0.113*	
H10C	0.7861	0.0787	0.1126	0.113*	
C11	0.7406 (3)	0.5875 (3)	0.2587 (2)	0.0380 (10)	
H11A	0.6689	0.5569	0.2674	0.046*	
H11B	0.7810	0.6123	0.3100	0.046*	
C12	0.7246 (3)	0.6668 (3)	0.2269 (2)	0.0368 (9)	
C13	0.6452 (3)	0.6588 (3)	0.1704 (2)	0.0372 (9)	
H13	0.5989	0.6017	0.1511	0.045*	
C14	0.6310 (3)	0.7304 (3)	0.1413 (3)	0.0468 (11)	
H14	0.5764	0.7225	0.1016	0.056*	
C15	0.6958 (4)	0.8137 (3)	0.1693 (3)	0.0557 (12)	
H15	0.6852	0.8640	0.1498	0.067*	
C16	0.7759 (4)	0.8245 (3)	0.2258 (3)	0.0569 (13)	
H16	0.8212	0.8820	0.2451	0.068*	
C17	0.7906 (3)	0.7510 (3)	0.2545 (3)	0.0507 (12)	
H17	0.8463	0.7584	0.2933	0.061*	
P1	0.81598 (7)	0.39475 (7)	0.47615 (6)	0.0315 (2)	
P2	0.96529 (8)	0.35871 (7)	0.59016 (6)	0.0327 (3)	
O3	0.5057 (3)	0.0855 (2)	0.2774 (2)	0.0756 (10)	
O4	0.75980 (18)	0.45926 (17)	0.53820 (13)	0.0344 (6)	
O5	0.88900 (19)	0.43548 (17)	0.42532 (15)	0.0393 (7)	
O6	0.88713 (19)	0.33499 (17)	0.51380 (14)	0.0378 (6)	
O7	0.9125 (2)	0.41021 (18)	0.66049 (14)	0.0418 (7)	
O8	1.07342 (19)	0.40236 (18)	0.57105 (16)	0.0457 (7)	
H8O	1.0923	0.4672	0.5723	0.055*	
O9	1.0373 (2)	−0.0103 (2)	0.61561 (18)	0.0569 (8)	
C18	0.9879 (3)	0.2457 (3)	0.5918 (2)	0.0345 (9)	
C19	0.9199 (3)	0.2000 (3)	0.6350 (2)	0.0461 (11)	
H19	0.8615	0.2289	0.6614	0.055*	
C20	0.9370 (3)	0.1143 (3)	0.6394 (3)	0.0504 (11)	
H20	0.8893	0.0835	0.6677	0.060*	
C21	1.0242 (3)	0.0721 (3)	0.6025 (2)	0.0429 (10)	
C22	1.0906 (3)	0.1155 (3)	0.5588 (2)	0.0410 (10)	
H22	1.1488	0.0864	0.5323	0.049*	
C23	1.0718 (3)	0.2009 (3)	0.5540 (2)	0.0339 (9)	
H23	1.1179	0.2302	0.5237	0.041*	
C24	1.1275 (4)	−0.0550 (3)	0.5836 (3)	0.0610 (13)	
H24A	1.1245	−0.0675	0.5266	0.091*	
H24B	1.1258	−0.1136	0.5964	0.091*	
H24C	1.1947	−0.0147	0.6059	0.091*	
C25	0.7237 (3)	0.3029 (3)	0.4153 (2)	0.0334 (9)	
C26	0.6180 (3)	0.2900 (3)	0.4363 (2)	0.0451 (11)	
H26	0.5963	0.3311	0.4833	0.054*	
C27	0.5422 (3)	0.2197 (3)	0.3916 (3)	0.0522 (12)	
H27	0.4695	0.2134	0.4072	0.063*	
C28	0.5735 (3)	0.1597 (3)	0.3251 (3)	0.0548 (12)	
C29	0.6787 (4)	0.1706 (3)	0.3021 (3)	0.0616 (14)	
H29	0.7002	0.1285	0.2555	0.074*	
C30	0.7522 (3)	0.2416 (3)	0.3460 (2)	0.0474 (11)	
H30	0.8239	0.2492	0.3288	0.057*	
C31	0.3922 (4)	0.0854 (4)	0.2906 (4)	0.108 (2)	
H31A	0.3779	0.0784	0.3426	0.162*	
H31B	0.3511	0.0336	0.2504	0.162*	
H31C	0.3698	0.1441	0.2878	0.162*	
S2	0.43158 (8)	0.51072 (7)	0.58904 (6)	0.0405 (3)	
O10	0.3239 (2)	0.1999 (2)	0.50801 (18)	0.0556 (8)	
O11	0.4524 (2)	0.1951 (2)	0.5948 (2)	0.0628 (9)	
N3	0.2994 (2)	0.3718 (3)	0.49185 (19)	0.0486 (9)	
H4N	0.2665	0.4166	0.4799	0.058*	
H5N	0.2806	0.3092	0.4787	0.058*	
N4	0.7141 (2)	0.5064 (2)	0.69929 (17)	0.0321 (7)	
H6N	0.7525	0.4945	0.6550	0.038*	
C32	0.6186 (3)	0.5523 (3)	0.6876 (2)	0.0354 (9)	
H32A	0.6394	0.5975	0.6584	0.042*	
H32B	0.5928	0.5864	0.7391	0.042*	
C33	0.5300 (3)	0.4802 (3)	0.6427 (2)	0.0324 (9)	
C34	0.5221 (3)	0.3891 (3)	0.6331 (2)	0.0330 (9)	
C35	0.6067 (3)	0.3500 (3)	0.6690 (2)	0.0392 (10)	
H35A	0.6507	0.3152	0.6272	0.047*	
H35B	0.5708	0.3058	0.6951	0.047*	
C36	0.6806 (3)	0.4253 (3)	0.7285 (2)	0.0378 (10)	
H36A	0.6424	0.4462	0.7779	0.045*	
H36B	0.7459	0.3994	0.7402	0.045*	
C37	0.3792 (3)	0.3946 (3)	0.5479 (2)	0.0367 (9)	
C38	0.4325 (3)	0.3380 (3)	0.5790 (2)	0.0354 (9)	
C39	0.3967 (3)	0.2406 (3)	0.5566 (3)	0.0465 (11)	
C40	0.4057 (10)	0.1022 (6)	0.5965 (10)	0.064 (3)	0.539 (18)
H40A	0.3314	0.1048	0.6158	0.077*	0.539 (18)
H40B	0.4008	0.0590	0.5425	0.077*	0.539 (18)
C41	0.4690 (15)	0.0685 (15)	0.6453 (11)	0.105 (5)	0.539 (18)
H41A	0.4358	0.0066	0.6452	0.157*	0.539 (18)
H41B	0.5422	0.0648	0.6258	0.157*	0.539 (18)
H41C	0.4729	0.1106	0.6989	0.157*	0.539 (18)
C40A	0.4284 (13)	0.0939 (4)	0.5608 (10)	0.064 (3)	0.461 (18)
H40C	0.3503	0.0732	0.5631	0.077*	0.461 (18)
H40D	0.4492	0.0743	0.5055	0.077*	0.461 (18)
C41A	0.4914 (17)	0.0572 (17)	0.6077 (13)	0.105 (5)	0.461 (18)
H41D	0.4797	−0.0107	0.5885	0.157*	0.461 (18)
H41E	0.5682	0.0791	0.6050	0.157*	0.461 (18)
H41F	0.4700	0.0779	0.6622	0.157*	0.461 (18)
C42	0.7991 (3)	0.5739 (3)	0.7553 (2)	0.0352 (9)	
H42A	0.7695	0.5932	0.8082	0.042*	
H42B	0.8630	0.5421	0.7589	0.042*	
C43	0.8340 (3)	0.6581 (3)	0.7306 (2)	0.0318 (9)	
C44	0.8986 (3)	0.6526 (3)	0.6664 (2)	0.0359 (9)	
H44	0.9210	0.5947	0.6384	0.043*	
C45	0.9305 (3)	0.7302 (3)	0.6430 (2)	0.0442 (11)	
H45	0.9744	0.7253	0.5990	0.053*	
C46	0.8991 (3)	0.8145 (3)	0.6830 (3)	0.0516 (12)	
H46	0.9199	0.8679	0.6666	0.062*	
C47	0.8363 (3)	0.8202 (3)	0.7480 (3)	0.0527 (12)	
H47	0.8152	0.8782	0.7768	0.063*	
C48	0.8045 (3)	0.7425 (3)	0.7710 (3)	0.0480 (11)	
H48	0.7616	0.7476	0.8155	0.058*	
P3	0.65068 (8)	0.41361 (7)	−0.02081 (6)	0.0346 (3)	
P4	0.51268 (8)	0.36322 (8)	0.10008 (6)	0.0396 (3)	
O12	0.3630 (5)	−0.0312 (3)	0.0713 (7)	0.076 (2)	0.82 (2)
O12A	0.349 (2)	−0.0292 (9)	0.041 (2)	0.076 (2)	0.18 (2)
O13	0.5969 (2)	0.4040 (2)	0.16197 (16)	0.0590 (9)	
O14	0.4091 (2)	0.4079 (2)	0.10712 (18)	0.0581 (9)	
H14O	0.4041	0.4601	0.0861	0.070*	
O15	0.5546 (2)	0.3624 (2)	0.01597 (15)	0.0497 (8)	
O16	0.5953 (2)	0.45006 (19)	−0.07756 (16)	0.0482 (7)	
O17	0.72531 (19)	0.47797 (17)	0.04102 (14)	0.0401 (7)	
O18	0.8492 (3)	0.0875 (2)	−0.20452 (18)	0.0606 (9)	
C49	0.4671 (3)	0.2452 (3)	0.0909 (2)	0.0446 (10)	
C50	0.3649 (3)	0.2030 (3)	0.0589 (2)	0.0502 (11)	
H50	0.3176	0.2384	0.0416	0.060*	
C51	0.3306 (4)	0.1113 (3)	0.0518 (3)	0.0626 (14)	
H51	0.2600	0.0839	0.0306	0.075*	
C52	0.3998 (4)	0.0598 (3)	0.0759 (3)	0.0643 (14)	
C53	0.5025 (4)	0.1000 (4)	0.1085 (3)	0.0756 (16)	
H53	0.5499	0.0642	0.1252	0.091*	
C54	0.5344 (4)	0.1923 (3)	0.1162 (3)	0.0635 (14)	
H54	0.6039	0.2203	0.1393	0.076*	
C55	0.4393 (10)	−0.0872 (6)	0.0903 (8)	0.079 (3)	0.82 (2)
H55A	0.4026	−0.1497	0.0847	0.118*	0.82 (2)
H55B	0.4992	−0.0910	0.0546	0.118*	0.82 (2)
H55C	0.4677	−0.0587	0.1445	0.118*	0.82 (2)
C55A	0.398 (4)	−0.1063 (10)	0.052 (3)	0.079 (3)	0.18 (2)
H55D	0.3534	−0.1647	0.0242	0.118*	0.18 (2)
H55E	0.4709	−0.1059	0.0326	0.118*	0.18 (2)
H55F	0.4013	−0.1004	0.1086	0.118*	0.18 (2)
C56	0.7129 (3)	0.3193 (3)	−0.0772 (2)	0.0334 (9)	
C57	0.7997 (3)	0.2912 (3)	−0.0458 (2)	0.0492 (11)	
H57	0.8264	0.3247	0.0060	0.059*	
C58	0.8488 (4)	0.2162 (3)	−0.0871 (3)	0.0546 (12)	
H58	0.9098	0.1996	−0.0647	0.065*	
C59	0.8091 (3)	0.1660 (3)	−0.1607 (3)	0.0460 (11)	
C60	0.7253 (3)	0.1931 (3)	−0.1958 (3)	0.0502 (11)	
H60	0.7010	0.1602	−0.2482	0.060*	
C61	0.6770 (3)	0.2689 (3)	−0.1535 (2)	0.0453 (11)	
H61	0.6180	0.2869	−0.1771	0.054*	
C62	0.9472 (4)	0.0671 (4)	−0.1766 (3)	0.0776 (16)	
H62A	1.0019	0.1219	−0.1659	0.116*	
H62B	0.9729	0.0153	−0.2163	0.116*	
H62C	0.9347	0.0501	−0.1282	0.116*	

### Atomic displacement parameters (Å^2^)

**Table:**

	*U*^11^	*U*^22^	*U*^33^	*U*^12^	*U*^13^	*U*^23^
S1	0.0297 (5)	0.0417 (6)	0.0369 (6)	−0.0015 (4)	0.0015 (4)	0.0143 (5)
O1	0.0394 (16)	0.0458 (18)	0.0550 (19)	0.0107 (14)	0.0074 (14)	0.0139 (15)
O2	0.0444 (17)	0.0361 (17)	0.0517 (18)	0.0035 (13)	0.0110 (14)	0.0142 (14)
N1	0.0309 (16)	0.0327 (18)	0.0236 (17)	0.0047 (13)	0.0015 (13)	0.0037 (14)
N2	0.0314 (18)	0.048 (2)	0.051 (2)	0.0002 (16)	0.0116 (16)	0.0151 (18)
C1	0.0270 (19)	0.035 (2)	0.037 (2)	−0.0014 (16)	−0.0005 (16)	0.0126 (18)
C2	0.0240 (18)	0.039 (2)	0.026 (2)	0.0007 (16)	−0.0005 (15)	0.0096 (17)
C3	0.0253 (19)	0.037 (2)	0.026 (2)	0.0004 (16)	−0.0018 (16)	0.0089 (17)
C4	0.034 (2)	0.034 (2)	0.036 (2)	0.0030 (17)	0.0009 (17)	0.0139 (19)
C5	0.037 (2)	0.040 (2)	0.036 (2)	0.0028 (18)	0.0026 (18)	0.0134 (19)
C6	0.027 (2)	0.043 (2)	0.032 (2)	−0.0007 (17)	−0.0028 (17)	0.0117 (19)
C7	0.0279 (19)	0.032 (2)	0.030 (2)	0.0034 (16)	−0.0028 (16)	0.0078 (17)
C8	0.029 (2)	0.048 (3)	0.034 (2)	0.0037 (19)	−0.0006 (18)	0.013 (2)
C9	0.062 (3)	0.038 (3)	0.066 (3)	0.007 (2)	0.014 (2)	0.019 (2)
C10	0.077 (4)	0.055 (3)	0.099 (4)	0.008 (3)	0.022 (3)	0.030 (3)
C11	0.034 (2)	0.043 (2)	0.034 (2)	0.0089 (18)	0.0067 (18)	0.0064 (19)
C12	0.035 (2)	0.037 (2)	0.033 (2)	0.0039 (18)	0.0015 (18)	0.0013 (19)
C13	0.027 (2)	0.040 (2)	0.039 (2)	0.0028 (17)	−0.0007 (18)	0.0030 (19)
C14	0.037 (2)	0.051 (3)	0.047 (3)	0.006 (2)	−0.009 (2)	0.008 (2)
C15	0.053 (3)	0.056 (3)	0.062 (3)	0.012 (2)	−0.007 (2)	0.023 (3)
C16	0.053 (3)	0.039 (3)	0.071 (3)	−0.007 (2)	−0.018 (2)	0.011 (2)
C17	0.046 (3)	0.042 (3)	0.056 (3)	0.002 (2)	−0.018 (2)	0.004 (2)
P1	0.0279 (5)	0.0380 (6)	0.0283 (6)	0.0013 (4)	0.0033 (4)	0.0104 (5)
P2	0.0284 (5)	0.0374 (6)	0.0313 (6)	0.0006 (4)	−0.0003 (4)	0.0098 (5)
O3	0.056 (2)	0.067 (2)	0.082 (3)	−0.0112 (17)	−0.0132 (18)	−0.006 (2)
O4	0.0329 (14)	0.0387 (15)	0.0296 (15)	0.0043 (12)	0.0023 (11)	0.0073 (12)
O5	0.0403 (15)	0.0363 (16)	0.0406 (16)	−0.0009 (12)	0.0136 (12)	0.0124 (13)
O6	0.0369 (15)	0.0428 (16)	0.0299 (15)	0.0093 (12)	−0.0099 (11)	0.0038 (12)
O7	0.0520 (17)	0.0471 (17)	0.0269 (15)	0.0148 (13)	0.0062 (12)	0.0084 (13)
O8	0.0289 (14)	0.0345 (16)	0.074 (2)	−0.0029 (12)	0.0070 (14)	0.0202 (15)
O9	0.062 (2)	0.0410 (18)	0.073 (2)	0.0013 (15)	−0.0035 (17)	0.0273 (17)
C18	0.027 (2)	0.042 (2)	0.034 (2)	−0.0042 (17)	−0.0038 (17)	0.0131 (19)
C19	0.048 (3)	0.049 (3)	0.048 (3)	0.004 (2)	0.011 (2)	0.024 (2)
C20	0.048 (3)	0.052 (3)	0.057 (3)	0.000 (2)	0.010 (2)	0.028 (2)
C21	0.042 (2)	0.037 (2)	0.049 (3)	−0.0031 (19)	−0.007 (2)	0.015 (2)
C22	0.035 (2)	0.038 (2)	0.047 (3)	0.0054 (19)	0.0002 (19)	0.008 (2)
C23	0.032 (2)	0.037 (2)	0.031 (2)	−0.0003 (17)	−0.0016 (17)	0.0086 (18)
C24	0.060 (3)	0.043 (3)	0.082 (4)	0.007 (2)	−0.006 (3)	0.023 (3)
C25	0.030 (2)	0.039 (2)	0.033 (2)	0.0075 (17)	−0.0016 (17)	0.0126 (19)
C26	0.039 (2)	0.054 (3)	0.037 (2)	−0.002 (2)	0.0070 (19)	0.007 (2)
C27	0.037 (2)	0.052 (3)	0.061 (3)	−0.005 (2)	0.002 (2)	0.012 (3)
C28	0.038 (3)	0.047 (3)	0.065 (3)	−0.001 (2)	−0.012 (2)	−0.001 (3)
C29	0.052 (3)	0.066 (3)	0.049 (3)	0.011 (2)	−0.003 (2)	−0.012 (3)
C30	0.033 (2)	0.059 (3)	0.041 (3)	0.005 (2)	0.0021 (19)	0.002 (2)
C31	0.048 (3)	0.093 (5)	0.148 (6)	−0.011 (3)	−0.036 (4)	−0.009 (4)
S2	0.0326 (5)	0.0500 (7)	0.0409 (6)	0.0071 (5)	0.0018 (4)	0.0157 (5)
O10	0.0359 (17)	0.0538 (19)	0.069 (2)	−0.0069 (14)	−0.0044 (15)	0.0118 (17)
O11	0.0513 (19)	0.0429 (19)	0.093 (3)	−0.0065 (15)	−0.0149 (18)	0.0240 (18)
N3	0.0350 (19)	0.060 (2)	0.051 (2)	0.0039 (17)	−0.0044 (17)	0.0177 (19)
N4	0.0273 (16)	0.0392 (19)	0.0274 (17)	0.0012 (14)	0.0036 (13)	0.0073 (15)
C32	0.035 (2)	0.040 (2)	0.031 (2)	0.0056 (18)	0.0025 (17)	0.0105 (19)
C33	0.0243 (19)	0.047 (3)	0.026 (2)	0.0040 (17)	0.0053 (16)	0.0110 (18)
C34	0.0242 (19)	0.045 (3)	0.031 (2)	0.0038 (17)	0.0115 (16)	0.0134 (19)
C35	0.035 (2)	0.040 (2)	0.044 (3)	−0.0011 (18)	0.0030 (19)	0.017 (2)
C36	0.039 (2)	0.045 (2)	0.033 (2)	0.0029 (19)	0.0047 (18)	0.018 (2)
C37	0.0218 (19)	0.052 (3)	0.036 (2)	0.0009 (18)	0.0056 (17)	0.014 (2)
C38	0.025 (2)	0.041 (2)	0.038 (2)	0.0003 (17)	0.0081 (17)	0.0108 (19)
C39	0.029 (2)	0.052 (3)	0.058 (3)	0.001 (2)	0.006 (2)	0.017 (2)
C40	0.068 (4)	0.050 (3)	0.075 (6)	0.000 (3)	−0.002 (4)	0.023 (4)
C41	0.120 (7)	0.082 (6)	0.121 (11)	−0.001 (5)	−0.017 (8)	0.050 (8)
C40A	0.068 (4)	0.050 (3)	0.075 (6)	0.000 (3)	−0.002 (4)	0.023 (4)
C41A	0.120 (7)	0.082 (6)	0.121 (11)	−0.001 (5)	−0.017 (8)	0.050 (8)
C42	0.030 (2)	0.042 (2)	0.028 (2)	0.0026 (17)	−0.0011 (17)	0.0032 (18)
C43	0.0256 (19)	0.038 (2)	0.030 (2)	0.0041 (17)	0.0022 (16)	0.0080 (18)
C44	0.028 (2)	0.037 (2)	0.038 (2)	0.0008 (17)	0.0004 (17)	0.0041 (19)
C45	0.033 (2)	0.054 (3)	0.042 (3)	−0.007 (2)	0.0002 (19)	0.014 (2)
C46	0.046 (3)	0.047 (3)	0.061 (3)	−0.007 (2)	0.005 (2)	0.019 (2)
C47	0.046 (3)	0.043 (3)	0.064 (3)	0.006 (2)	0.008 (2)	0.009 (2)
C48	0.039 (2)	0.050 (3)	0.049 (3)	0.002 (2)	0.011 (2)	0.008 (2)
P3	0.0269 (5)	0.0370 (6)	0.0379 (6)	−0.0002 (4)	0.0000 (4)	0.0097 (5)
P4	0.0383 (6)	0.0457 (7)	0.0347 (6)	0.0015 (5)	0.0038 (5)	0.0134 (5)
O12	0.089 (3)	0.050 (2)	0.087 (6)	−0.009 (2)	0.035 (3)	0.024 (2)
O12A	0.089 (3)	0.050 (2)	0.087 (6)	−0.009 (2)	0.035 (3)	0.024 (2)
O13	0.068 (2)	0.064 (2)	0.0386 (17)	−0.0166 (16)	−0.0178 (15)	0.0158 (16)
O14	0.0464 (18)	0.062 (2)	0.079 (2)	0.0203 (15)	0.0286 (16)	0.0361 (18)
O15	0.0375 (16)	0.067 (2)	0.0301 (15)	−0.0156 (14)	0.0085 (12)	0.0002 (14)
O16	0.0505 (17)	0.0493 (18)	0.0469 (18)	0.0084 (14)	−0.0053 (14)	0.0170 (15)
O17	0.0366 (15)	0.0414 (16)	0.0359 (16)	−0.0056 (12)	0.0015 (12)	0.0057 (13)
O18	0.061 (2)	0.054 (2)	0.059 (2)	0.0113 (16)	0.0069 (17)	0.0026 (17)
C49	0.043 (2)	0.045 (3)	0.045 (3)	−0.001 (2)	0.004 (2)	0.014 (2)
C50	0.038 (2)	0.061 (3)	0.052 (3)	0.005 (2)	0.008 (2)	0.017 (2)
C51	0.048 (3)	0.048 (3)	0.082 (4)	−0.007 (2)	0.017 (3)	0.009 (3)
C52	0.064 (3)	0.044 (3)	0.081 (4)	−0.003 (3)	0.030 (3)	0.016 (3)
C53	0.061 (3)	0.063 (4)	0.114 (5)	0.001 (3)	0.006 (3)	0.046 (3)
C54	0.046 (3)	0.063 (3)	0.092 (4)	0.004 (2)	−0.002 (3)	0.041 (3)
C55	0.096 (6)	0.048 (4)	0.095 (7)	0.004 (4)	0.034 (5)	0.025 (4)
C55A	0.096 (6)	0.048 (4)	0.095 (7)	0.004 (4)	0.034 (5)	0.025 (4)
C56	0.029 (2)	0.037 (2)	0.031 (2)	−0.0015 (17)	0.0000 (17)	0.0065 (18)
C57	0.052 (3)	0.046 (3)	0.043 (3)	0.010 (2)	−0.012 (2)	0.000 (2)
C58	0.058 (3)	0.051 (3)	0.053 (3)	0.019 (2)	−0.012 (2)	0.008 (2)
C59	0.049 (3)	0.044 (3)	0.043 (3)	0.009 (2)	0.005 (2)	0.009 (2)
C60	0.050 (3)	0.051 (3)	0.042 (3)	0.001 (2)	−0.002 (2)	0.004 (2)
C61	0.039 (2)	0.057 (3)	0.039 (3)	0.001 (2)	−0.0034 (19)	0.016 (2)
C62	0.081 (4)	0.060 (3)	0.083 (4)	0.025 (3)	−0.007 (3)	0.002 (3)

### Geometric parameters (Å, º)

**Table:**

S1—C6	1.730 (4)	N3—C37	1.345 (4)
S1—C2	1.735 (3)	N3—H4N	0.9099
O1—C8	1.229 (4)	N3—H5N	0.9099
O2—C8	1.347 (4)	N4—C36	1.491 (4)
O2—C9	1.451 (5)	N4—C32	1.492 (4)
N1—C1	1.494 (4)	N4—C42	1.515 (4)
N1—C5	1.502 (4)	N4—H6N	0.9100
N1—C11	1.511 (4)	C32—C33	1.497 (5)
N1—H1N	0.9100	C32—H32A	0.9900
N2—C6	1.348 (4)	C32—H32B	0.9900
N2—H2N	0.9100	C33—C34	1.335 (5)
N2—H3N	0.9101	C34—C38	1.455 (5)
C1—C2	1.497 (5)	C34—C35	1.495 (5)
C1—H1A	0.9900	C35—C36	1.518 (5)
C1—H1B	0.9900	C35—H35A	0.9900
C2—C3	1.335 (5)	C35—H35B	0.9900
C3—C7	1.449 (5)	C36—H36A	0.9900
C3—C4	1.506 (5)	C36—H36B	0.9900
C4—C5	1.510 (5)	C37—C38	1.378 (5)
C4—H4A	0.9900	C38—C39	1.428 (5)
C4—H4B	0.9900	C40—C41	1.413 (8)
C5—H5A	0.9900	C40—H40A	0.9900
C5—H5B	0.9900	C40—H40B	0.9900
C6—C7	1.388 (5)	C41—H41A	0.9800
C7—C8	1.429 (5)	C41—H41B	0.9800
C9—C10	1.487 (6)	C41—H41C	0.9800
C9—H9A	0.9900	C40A—C41A	1.414 (8)
C9—H9B	0.9900	C40A—H40C	0.9900
C10—H10A	0.9800	C40A—H40D	0.9900
C10—H10B	0.9800	C41A—H41D	0.9800
C10—H10C	0.9800	C41A—H41E	0.9800
C11—C12	1.507 (5)	C41A—H41F	0.9800
C11—H11A	0.9900	C42—C43	1.491 (5)
C11—H11B	0.9900	C42—H42A	0.9900
C12—C13	1.377 (5)	C42—H42B	0.9900
C12—C17	1.388 (5)	C43—C48	1.369 (5)
C13—C14	1.365 (5)	C43—C44	1.393 (5)
C13—H13	0.9500	C44—C45	1.378 (5)
C14—C15	1.371 (6)	C44—H44	0.9500
C14—H14	0.9500	C45—C46	1.375 (6)
C15—C16	1.374 (6)	C45—H45	0.9500
C15—H15	0.9500	C46—C47	1.391 (6)
C16—C17	1.388 (6)	C46—H46	0.9500
C16—H16	0.9500	C47—C48	1.375 (6)
C17—H17	0.9500	C47—H47	0.9500
P1—O4	1.488 (2)	C48—H48	0.9500
P1—O5	1.496 (2)	P3—O17	1.473 (3)
P1—O6	1.614 (3)	P3—O16	1.490 (3)
P1—C25	1.776 (4)	P3—O15	1.598 (3)
P2—O7	1.476 (3)	P3—C56	1.759 (4)
P2—O8	1.514 (2)	P4—O13	1.460 (3)
P2—O6	1.595 (2)	P4—O14	1.517 (3)
P2—C18	1.781 (4)	P4—O15	1.590 (3)
O3—C28	1.380 (5)	P4—C49	1.774 (4)
O3—C31	1.433 (6)	O12—C52	1.383 (6)
O8—H8O	0.98	O12—C55	1.448 (7)
O9—C21	1.368 (5)	O12A—C52	1.383 (7)
O9—C24	1.417 (5)	O12A—C55A	1.448 (8)
C18—C23	1.386 (5)	O14—H14O	0.98
C18—C19	1.405 (5)	O18—C59	1.372 (5)
C19—C20	1.369 (5)	O18—C62	1.417 (5)
C19—H19	0.9500	C49—C54	1.386 (6)
C20—C21	1.397 (6)	C49—C50	1.392 (5)
C20—H20	0.9500	C50—C51	1.375 (6)
C21—C22	1.380 (5)	C50—H50	0.9500
C22—C23	1.374 (5)	C51—C52	1.375 (7)
C22—H22	0.9500	C51—H51	0.9500
C23—H23	0.9500	C52—C53	1.393 (7)
C24—H24A	0.9800	C53—C54	1.377 (6)
C24—H24B	0.9800	C53—H53	0.9500
C24—H24C	0.9800	C54—H54	0.9500
C25—C26	1.378 (5)	C55—H55A	0.9800
C25—C30	1.389 (5)	C55—H55B	0.9800
C26—C27	1.384 (5)	C55—H55C	0.9800
C26—H26	0.9500	C55A—H55D	0.9800
C27—C28	1.361 (6)	C55A—H55E	0.9800
C27—H27	0.9500	C55A—H55F	0.9800
C28—C29	1.380 (6)	C56—C57	1.381 (5)
C29—C30	1.368 (6)	C56—C61	1.393 (5)
C29—H29	0.9500	C57—C58	1.377 (6)
C30—H30	0.9500	C57—H57	0.9500
C31—H31A	0.9800	C58—C59	1.366 (6)
C31—H31B	0.9800	C58—H58	0.9500
C31—H31C	0.9800	C59—C60	1.376 (6)
S2—C33	1.737 (4)	C60—C61	1.386 (6)
S2—C37	1.742 (4)	C60—H60	0.9500
O10—C39	1.222 (5)	C61—H61	0.9500
O11—C39	1.346 (5)	C62—H62A	0.9800
O11—C40	1.468 (6)	C62—H62B	0.9800
O11—C40A	1.470 (6)	C62—H62C	0.9800
			
C6—S1—C2	90.77 (18)	C42—N4—H6N	98.9
C8—O2—C9	115.5 (3)	N4—C32—C33	109.2 (3)
C1—N1—C5	109.7 (3)	N4—C32—H32A	109.8
C1—N1—C11	112.5 (3)	C33—C32—H32A	109.8
C5—N1—C11	109.6 (3)	N4—C32—H32B	109.8
C1—N1—H1N	103.2	C33—C32—H32B	109.8
C5—N1—H1N	113.2	H32A—C32—H32B	108.3
C11—N1—H1N	108.6	C34—C33—C32	126.3 (3)
C6—N2—H2N	112.5	C34—C33—S2	113.5 (3)
C6—N2—H3N	125.1	C32—C33—S2	119.9 (3)
H2N—N2—H3N	120.9	C33—C34—C38	111.8 (3)
N1—C1—C2	109.0 (3)	C33—C34—C35	120.4 (3)
N1—C1—H1A	109.9	C38—C34—C35	127.5 (4)
C2—C1—H1A	109.9	C34—C35—C36	112.0 (3)
N1—C1—H1B	109.9	C34—C35—H35A	109.2
C2—C1—H1B	109.9	C36—C35—H35A	109.2
H1A—C1—H1B	108.3	C34—C35—H35B	109.2
C3—C2—C1	127.0 (3)	C36—C35—H35B	109.2
C3—C2—S1	113.3 (3)	H35A—C35—H35B	107.9
C1—C2—S1	119.7 (3)	N4—C36—C35	111.7 (3)
C2—C3—C7	112.6 (3)	N4—C36—H36A	109.3
C2—C3—C4	119.7 (3)	C35—C36—H36A	109.3
C7—C3—C4	127.6 (3)	N4—C36—H36B	109.3
C3—C4—C5	111.5 (3)	C35—C36—H36B	109.3
C3—C4—H4A	109.3	H36A—C36—H36B	107.9
C5—C4—H4A	109.3	N3—C37—C38	129.5 (4)
C3—C4—H4B	109.3	N3—C37—S2	119.5 (3)
C5—C4—H4B	109.3	C38—C37—S2	111.0 (3)
H4A—C4—H4B	108.0	C37—C38—C39	119.5 (4)
N1—C5—C4	111.6 (3)	C37—C38—C34	112.6 (3)
N1—C5—H5A	109.3	C39—C38—C34	127.9 (4)
C4—C5—H5A	109.3	O10—C39—O11	121.5 (4)
N1—C5—H5B	109.3	O10—C39—C38	125.7 (4)
C4—C5—H5B	109.3	O11—C39—C38	112.8 (4)
H5A—C5—H5B	108.0	C41—C40—O11	111.5 (11)
N2—C6—C7	128.6 (4)	C41—C40—H40A	109.3
N2—C6—S1	119.4 (3)	O11—C40—H40A	109.3
C7—C6—S1	111.9 (3)	C41—C40—H40B	109.3
C6—C7—C8	120.3 (3)	O11—C40—H40B	109.3
C6—C7—C3	111.3 (3)	H40A—C40—H40B	108.0
C8—C7—C3	128.4 (3)	C40—C41—H41A	109.5
O1—C8—O2	121.5 (4)	C40—C41—H41B	109.5
O1—C8—C7	125.3 (4)	H41A—C41—H41B	109.5
O2—C8—C7	113.2 (3)	C40—C41—H41C	109.5
O2—C9—C10	106.6 (4)	H41A—C41—H41C	109.5
O2—C9—H9A	110.4	H41B—C41—H41C	109.5
C10—C9—H9A	110.4	C41A—C40A—O11	104.6 (13)
O2—C9—H9B	110.4	C41A—C40A—H40C	110.8
C10—C9—H9B	110.4	O11—C40A—H40C	110.8
H9A—C9—H9B	108.6	C41A—C40A—H40D	110.8
C9—C10—H10A	109.5	O11—C40A—H40D	110.8
C9—C10—H10B	109.5	H40C—C40A—H40D	108.9
H10A—C10—H10B	109.5	C40A—C41A—H41D	109.5
C9—C10—H10C	109.5	C40A—C41A—H41E	109.5
H10A—C10—H10C	109.5	H41D—C41A—H41E	109.5
H10B—C10—H10C	109.5	C40A—C41A—H41F	109.5
C12—C11—N1	112.5 (3)	H41D—C41A—H41F	109.5
C12—C11—H11A	109.1	H41E—C41A—H41F	109.5
N1—C11—H11A	109.1	C43—C42—N4	112.7 (3)
C12—C11—H11B	109.1	C43—C42—H42A	109.1
N1—C11—H11B	109.1	N4—C42—H42A	109.1
H11A—C11—H11B	107.8	C43—C42—H42B	109.1
C13—C12—C17	118.0 (4)	N4—C42—H42B	109.1
C13—C12—C11	121.8 (3)	H42A—C42—H42B	107.8
C17—C12—C11	120.2 (4)	C48—C43—C44	118.6 (4)
C14—C13—C12	121.8 (4)	C48—C43—C42	120.8 (3)
C14—C13—H13	119.1	C44—C43—C42	120.6 (3)
C12—C13—H13	119.1	C45—C44—C43	120.8 (4)
C13—C14—C15	120.0 (4)	C45—C44—H44	119.6
C13—C14—H14	120.0	C43—C44—H44	119.6
C15—C14—H14	120.0	C46—C45—C44	120.3 (4)
C14—C15—C16	119.9 (4)	C46—C45—H45	119.8
C14—C15—H15	120.1	C44—C45—H45	119.8
C16—C15—H15	120.1	C45—C46—C47	118.8 (4)
C15—C16—C17	119.8 (4)	C45—C46—H46	120.6
C15—C16—H16	120.1	C47—C46—H46	120.6
C17—C16—H16	120.1	C48—C47—C46	120.6 (4)
C16—C17—C12	120.5 (4)	C48—C47—H47	119.7
C16—C17—H17	119.7	C46—C47—H47	119.7
C12—C17—H17	119.7	C43—C48—C47	120.8 (4)
O4—P1—O5	117.86 (15)	C43—C48—H48	119.6
O4—P1—O6	110.63 (14)	C47—C48—H48	119.6
O5—P1—O6	106.79 (14)	O17—P3—O16	119.32 (16)
O4—P1—C25	111.79 (16)	O17—P3—O15	111.18 (15)
O5—P1—C25	108.85 (16)	O16—P3—O15	104.61 (16)
O6—P1—C25	99.20 (15)	O17—P3—C56	111.96 (16)
O7—P2—O8	117.93 (16)	O16—P3—C56	106.39 (17)
O7—P2—O6	111.01 (14)	O15—P3—C56	101.71 (16)
O8—P2—O6	107.02 (15)	O13—P4—O14	117.31 (19)
O7—P2—C18	113.03 (16)	O13—P4—O15	112.12 (16)
O8—P2—C18	104.90 (16)	O14—P4—O15	105.06 (17)
O6—P2—C18	101.47 (15)	O13—P4—C49	112.75 (19)
C28—O3—C31	116.2 (4)	O14—P4—C49	103.79 (18)
P2—O6—P1	134.33 (17)	O15—P4—C49	104.65 (18)
P2—O8—H8O	126.4	C52—O12—C55	118.2 (5)
C21—O9—C24	118.3 (3)	C52—O12A—C55A	118.3 (8)
C23—C18—C19	117.7 (4)	P4—O14—H14O	120.0
C23—C18—P2	123.1 (3)	P4—O15—P3	138.67 (18)
C19—C18—P2	119.2 (3)	C59—O18—C62	117.2 (4)
C20—C19—C18	120.7 (4)	C54—C49—C50	118.1 (4)
C20—C19—H19	119.6	C54—C49—P4	119.8 (3)
C18—C19—H19	119.6	C50—C49—P4	122.1 (3)
C19—C20—C21	120.2 (4)	C51—C50—C49	121.6 (4)
C19—C20—H20	119.9	C51—C50—H50	119.2
C21—C20—H20	119.9	C49—C50—H50	119.2
O9—C21—C22	125.4 (4)	C50—C51—C52	119.1 (5)
O9—C21—C20	114.9 (4)	C50—C51—H51	120.4
C22—C21—C20	119.7 (4)	C52—C51—H51	120.4
C23—C22—C21	119.5 (4)	C51—C52—O12	118.6 (6)
C23—C22—H22	120.3	C51—C52—O12A	100.8 (14)
C21—C22—H22	120.3	C51—C52—C53	120.8 (5)
C22—C23—C18	122.1 (4)	O12—C52—C53	120.4 (6)
C22—C23—H23	119.0	O12A—C52—C53	136.3 (14)
C18—C23—H23	119.0	C54—C53—C52	119.0 (5)
O9—C24—H24A	109.5	C54—C53—H53	120.5
O9—C24—H24B	109.5	C52—C53—H53	120.5
H24A—C24—H24B	109.5	C53—C54—C49	121.3 (5)
O9—C24—H24C	109.5	C53—C54—H54	119.4
H24A—C24—H24C	109.5	C49—C54—H54	119.4
H24B—C24—H24C	109.5	O12—C55—H55A	109.5
C26—C25—C30	117.0 (4)	O12—C55—H55B	109.5
C26—C25—P1	119.7 (3)	H55A—C55—H55B	109.5
C30—C25—P1	123.3 (3)	O12—C55—H55C	109.5
C25—C26—C27	122.6 (4)	H55A—C55—H55C	109.5
C25—C26—H26	118.7	H55B—C55—H55C	109.5
C27—C26—H26	118.7	O12A—C55A—H55D	109.5
C28—C27—C26	118.9 (4)	O12A—C55A—H55E	109.5
C28—C27—H27	120.6	H55D—C55A—H55E	109.5
C26—C27—H27	120.6	O12A—C55A—H55F	109.5
C27—C28—C29	120.1 (4)	H55D—C55A—H55F	109.5
C27—C28—O3	123.8 (4)	H55E—C55A—H55F	109.5
C29—C28—O3	116.1 (4)	C57—C56—C61	117.1 (4)
C30—C29—C28	120.3 (4)	C57—C56—P3	120.2 (3)
C30—C29—H29	119.8	C61—C56—P3	122.6 (3)
C28—C29—H29	119.8	C58—C57—C56	122.1 (4)
C29—C30—C25	121.1 (4)	C58—C57—H57	119.0
C29—C30—H30	119.5	C56—C57—H57	119.0
C25—C30—H30	119.5	C59—C58—C57	119.4 (4)
O3—C31—H31A	109.5	C59—C58—H58	120.3
O3—C31—H31B	109.5	C57—C58—H58	120.3
H31A—C31—H31B	109.5	C58—C59—O18	123.4 (4)
O3—C31—H31C	109.5	C58—C59—C60	120.7 (4)
H31A—C31—H31C	109.5	O18—C59—C60	115.8 (4)
H31B—C31—H31C	109.5	C59—C60—C61	119.0 (4)
C33—S2—C37	90.99 (19)	C59—C60—H60	120.5
C39—O11—C40	118.9 (5)	C61—C60—H60	120.5
C39—O11—C40A	111.8 (6)	C60—C61—C56	121.4 (4)
C37—N3—H4N	120.6	C60—C61—H61	119.3
C37—N3—H5N	107.5	C56—C61—H61	119.3
H4N—N3—H5N	130.9	O18—C62—H62A	109.5
C36—N4—C32	110.9 (3)	O18—C62—H62B	109.5
C36—N4—C42	110.0 (3)	H62A—C62—H62B	109.5
C32—N4—C42	111.4 (3)	O18—C62—H62C	109.5
C36—N4—H6N	115.8	H62A—C62—H62C	109.5
C32—N4—H6N	109.4	H62B—C62—H62C	109.5
			
C5—N1—C1—C2	48.7 (4)	C37—S2—C33—C34	−2.3 (3)
C11—N1—C1—C2	171.0 (3)	C37—S2—C33—C32	172.2 (3)
N1—C1—C2—C3	−16.7 (5)	C32—C33—C34—C38	−173.7 (3)
N1—C1—C2—S1	160.6 (2)	S2—C33—C34—C38	0.4 (4)
C6—S1—C2—C3	2.8 (3)	C32—C33—C34—C35	0.1 (6)
C6—S1—C2—C1	−174.8 (3)	S2—C33—C34—C35	174.2 (3)
C1—C2—C3—C7	175.7 (3)	C33—C34—C35—C36	12.8 (5)
S1—C2—C3—C7	−1.7 (4)	C38—C34—C35—C36	−174.6 (3)
C1—C2—C3—C4	−2.1 (6)	C32—N4—C36—C35	63.9 (4)
S1—C2—C3—C4	−179.6 (3)	C42—N4—C36—C35	−172.5 (3)
C2—C3—C4—C5	−12.1 (5)	C34—C35—C36—N4	−43.9 (4)
C7—C3—C4—C5	170.4 (3)	C33—S2—C37—N3	−174.6 (3)
C1—N1—C5—C4	−66.1 (4)	C33—S2—C37—C38	3.7 (3)
C11—N1—C5—C4	169.9 (3)	N3—C37—C38—C39	−6.1 (6)
C3—C4—C5—N1	45.4 (4)	S2—C37—C38—C39	175.9 (3)
C2—S1—C6—N2	176.2 (3)	N3—C37—C38—C34	173.9 (3)
C2—S1—C6—C7	−3.2 (3)	S2—C37—C38—C34	−4.1 (4)
N2—C6—C7—C8	0.5 (6)	C33—C34—C38—C37	2.4 (4)
S1—C6—C7—C8	179.8 (3)	C35—C34—C38—C37	−170.8 (3)
N2—C6—C7—C3	−176.5 (3)	C33—C34—C38—C39	−177.6 (4)
S1—C6—C7—C3	2.8 (4)	C35—C34—C38—C39	9.2 (6)
C2—C3—C7—C6	−0.7 (4)	C40—O11—C39—O10	−18.3 (10)
C4—C3—C7—C6	176.9 (3)	C40A—O11—C39—O10	10.4 (10)
C2—C3—C7—C8	−177.4 (4)	C40—O11—C39—C38	161.8 (8)
C4—C3—C7—C8	0.2 (6)	C40A—O11—C39—C38	−169.5 (9)
C9—O2—C8—O1	−0.2 (5)	C37—C38—C39—O10	3.6 (6)
C9—O2—C8—C7	179.0 (3)	C34—C38—C39—O10	−176.4 (4)
C6—C7—C8—O1	2.9 (6)	C37—C38—C39—O11	−176.6 (3)
C3—C7—C8—O1	179.3 (4)	C34—C38—C39—O11	3.5 (6)
C6—C7—C8—O2	−176.2 (3)	C39—O11—C40—C41	−174.1 (9)
C3—C7—C8—O2	0.3 (5)	C39—O11—C40A—C41A	−179.3 (10)
C8—O2—C9—C10	177.3 (4)	C36—N4—C42—C43	−178.4 (3)
C1—N1—C11—C12	55.5 (4)	C32—N4—C42—C43	−55.1 (4)
C5—N1—C11—C12	177.8 (3)	N4—C42—C43—C48	108.7 (4)
N1—C11—C12—C13	79.0 (4)	N4—C42—C43—C44	−72.2 (4)
N1—C11—C12—C17	−101.0 (4)	C48—C43—C44—C45	−1.3 (5)
C17—C12—C13—C14	0.4 (6)	C42—C43—C44—C45	179.6 (3)
C11—C12—C13—C14	−179.6 (4)	C43—C44—C45—C46	0.2 (6)
C12—C13—C14—C15	−1.2 (6)	C44—C45—C46—C47	1.0 (6)
C13—C14—C15—C16	1.1 (7)	C45—C46—C47—C48	−1.2 (6)
C14—C15—C16—C17	−0.4 (7)	C44—C43—C48—C47	1.1 (6)
C15—C16—C17—C12	−0.3 (7)	C42—C43—C48—C47	−179.7 (4)
C13—C12—C17—C16	0.3 (6)	C46—C47—C48—C43	0.1 (6)
C11—C12—C17—C16	−179.7 (4)	O13—P4—O15—P3	−19.5 (4)
O7—P2—O6—P1	50.0 (3)	O14—P4—O15—P3	109.0 (3)
O8—P2—O6—P1	−80.0 (3)	C49—P4—O15—P3	−142.0 (3)
C18—P2—O6—P1	170.3 (2)	O17—P3—O15—P4	3.2 (4)
O4—P1—O6—P2	−42.4 (3)	O16—P3—O15—P4	−126.9 (3)
O5—P1—O6—P2	87.0 (2)	C56—P3—O15—P4	122.5 (3)
C25—P1—O6—P2	−160.0 (2)	O13—P4—C49—C54	−24.3 (4)
O7—P2—C18—C23	−151.6 (3)	O14—P4—C49—C54	−152.3 (4)
O8—P2—C18—C23	−21.8 (4)	O15—P4—C49—C54	97.8 (4)
O6—P2—C18—C23	89.5 (3)	O13—P4—C49—C50	155.5 (3)
O7—P2—C18—C19	26.6 (4)	O14—P4—C49—C50	27.6 (4)
O8—P2—C18—C19	156.4 (3)	O15—P4—C49—C50	−82.3 (4)
O6—P2—C18—C19	−92.4 (3)	C54—C49—C50—C51	−0.4 (6)
C23—C18—C19—C20	0.2 (6)	P4—C49—C50—C51	179.7 (3)
P2—C18—C19—C20	−178.0 (3)	C49—C50—C51—C52	−1.0 (7)
C18—C19—C20—C21	1.6 (6)	C50—C51—C52—O12	177.5 (6)
C24—O9—C21—C22	1.5 (6)	C50—C51—C52—O12A	−164.6 (16)
C24—O9—C21—C20	−176.7 (4)	C50—C51—C52—C53	1.3 (7)
C19—C20—C21—O9	175.6 (4)	C55—O12—C52—C51	174.7 (5)
C19—C20—C21—C22	−2.7 (6)	C55—O12—C52—C53	−9.1 (9)
O9—C21—C22—C23	−176.3 (4)	C55A—O12A—C52—C51	−179 (3)
C20—C21—C22—C23	1.8 (6)	C55A—O12A—C52—C53	18 (4)
C21—C22—C23—C18	0.1 (6)	C51—C52—C53—C54	−0.3 (8)
C19—C18—C23—C22	−1.1 (6)	O12—C52—C53—C54	−176.4 (6)
P2—C18—C23—C22	177.1 (3)	O12A—C52—C53—C54	160 (2)
O4—P1—C25—C26	−10.2 (4)	C52—C53—C54—C49	−1.2 (8)
O5—P1—C25—C26	−142.1 (3)	C50—C49—C54—C53	1.5 (7)
O6—P1—C25—C26	106.5 (3)	P4—C49—C54—C53	−178.7 (4)
O4—P1—C25—C30	169.6 (3)	O17—P3—C56—C57	25.2 (4)
O5—P1—C25—C30	37.7 (4)	O16—P3—C56—C57	157.2 (3)
O6—P1—C25—C30	−73.7 (3)	O15—P3—C56—C57	−93.5 (3)
C30—C25—C26—C27	0.3 (6)	O17—P3—C56—C61	−156.1 (3)
P1—C25—C26—C27	−179.8 (3)	O16—P3—C56—C61	−24.1 (4)
C25—C26—C27—C28	1.2 (7)	O15—P3—C56—C61	85.2 (3)
C26—C27—C28—C29	−1.3 (7)	C61—C56—C57—C58	−0.6 (6)
C26—C27—C28—O3	178.0 (4)	P3—C56—C57—C58	178.2 (3)
C31—O3—C28—C27	13.9 (7)	C56—C57—C58—C59	−1.9 (7)
C31—O3—C28—C29	−166.7 (5)	C57—C58—C59—O18	−176.5 (4)
C27—C28—C29—C30	0.0 (8)	C57—C58—C59—C60	4.3 (7)
O3—C28—C29—C30	−179.4 (4)	C62—O18—C59—C58	−10.4 (6)
C28—C29—C30—C25	1.6 (7)	C62—O18—C59—C60	168.8 (4)
C26—C25—C30—C29	−1.7 (6)	C58—C59—C60—C61	−4.1 (7)
P1—C25—C30—C29	178.5 (4)	O18—C59—C60—C61	176.7 (4)
C36—N4—C32—C33	−48.0 (4)	C59—C60—C61—C56	1.5 (6)
C42—N4—C32—C33	−170.9 (3)	C57—C56—C61—C60	0.8 (6)
N4—C32—C33—C34	17.6 (5)	P3—C56—C61—C60	−177.9 (3)
N4—C32—C33—S2	−156.1 (2)		

### Hydrogen-bond geometry (Å, º)

**Table:**

*D*—H···*A*	*D*—H	H···*A*	*D*···*A*	*D*—H···*A*
N1—H1*N*···O13	0.91	2.37	2.853 (4)	113
N1—H1*N*···O17	0.91	2.06	2.924 (4)	159
N2—H2*N*···O1	0.91	2.07	2.767 (4)	132
N2—H3*N*···O17^i^	0.91	2.06	2.910 (4)	155
N3—H4*N*···O4^ii^	0.91	2.07	2.946 (5)	162
N3—H5*N*···O10	0.91	2.01	2.763 (6)	139
N4—H6*N*···O4	0.91	2.00	2.832 (4)	151
N4—H6*N*···O7	0.91	2.51	3.001 (4)	114
O8—H8*O*···O5^iii^	0.98	1.46	2.434 (4)	175
O14—H14*O*···O16^iv^	0.98	1.42	2.374 (4)	164
C1—H1*A*···O7^iii^	0.99	2.59	3.432 (4)	143
C5—H5*A*···O13	0.99	2.51	2.999 (5)	110
C11—H11*A*···O13	0.99	2.58	3.144 (5)	116
C14—H14···O15^iv^	0.95	2.57	3.466 (5)	158
C20—H20···O18^v^	0.95	2.32	3.119 (6)	141
C27—H27···O10	0.95	2.60	3.469 (5)	153
C36—H36*B*···O7	0.99	2.54	3.146 (5)	119
C41—H41*A*···O3^vi^	0.98	2.40	3.09 (2)	127
C42—H42*B*···O7	0.99	2.39	3.073 (5)	125
C46—H46···O9^vii^	0.95	2.60	3.508 (6)	161

Symmetry codes: (i) −*x*+2, −*y*+1, −*z*; (ii) −*x*+1, −*y*+1, −*z*+1; (iii) −*x*+2, −*y*+1, −*z*+1; (iv) −*x*+1, −*y*+1, −*z*; (v) *x*, *y*, *z*+1; (vi) −*x*+1, −*y*, −*z*+1; (vii) *x*, *y*+1, *z*.
