# Supplementary figures and images for: Crystal structure of (4-meth­oxy­phen­yl)[(4-meth­oxy­phen­yl)phospho­nato]dioxidophosphate(1−) 2-amino-6-benzyl-3-eth­oxy­carbon­yl-4,5,6,7-tetra­hydro­thieno[2,3-c]pyridin-6-ium
Source: Acta Crystallogr E Crystallogr Commun. 2015 Nov 28;71(Pt 12):o997–8. doi: 10.1107/S2056989015022331 (PMC4719942; doi:10.1107/S2056989015022331)

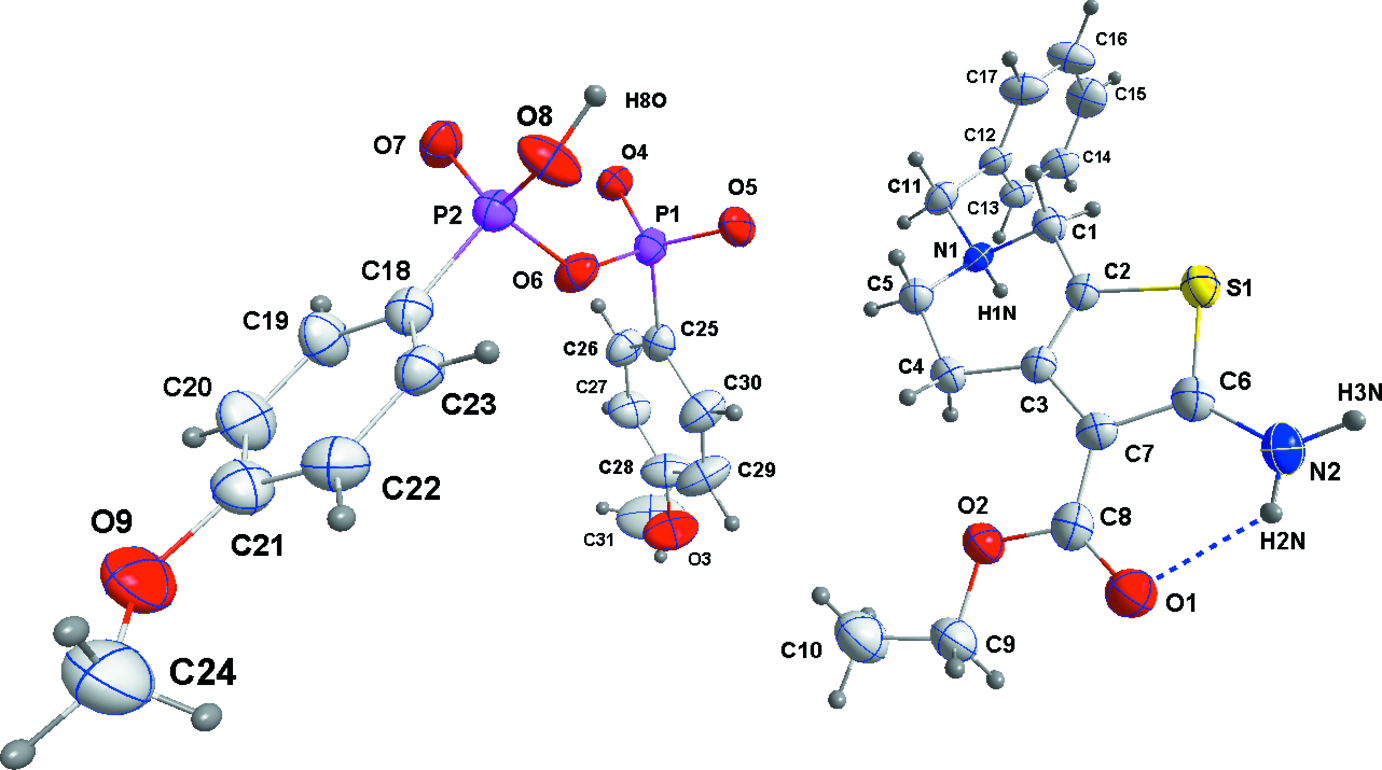

Supplement: Supplementary file 3 [file e-71-0o997-fig1.tif]

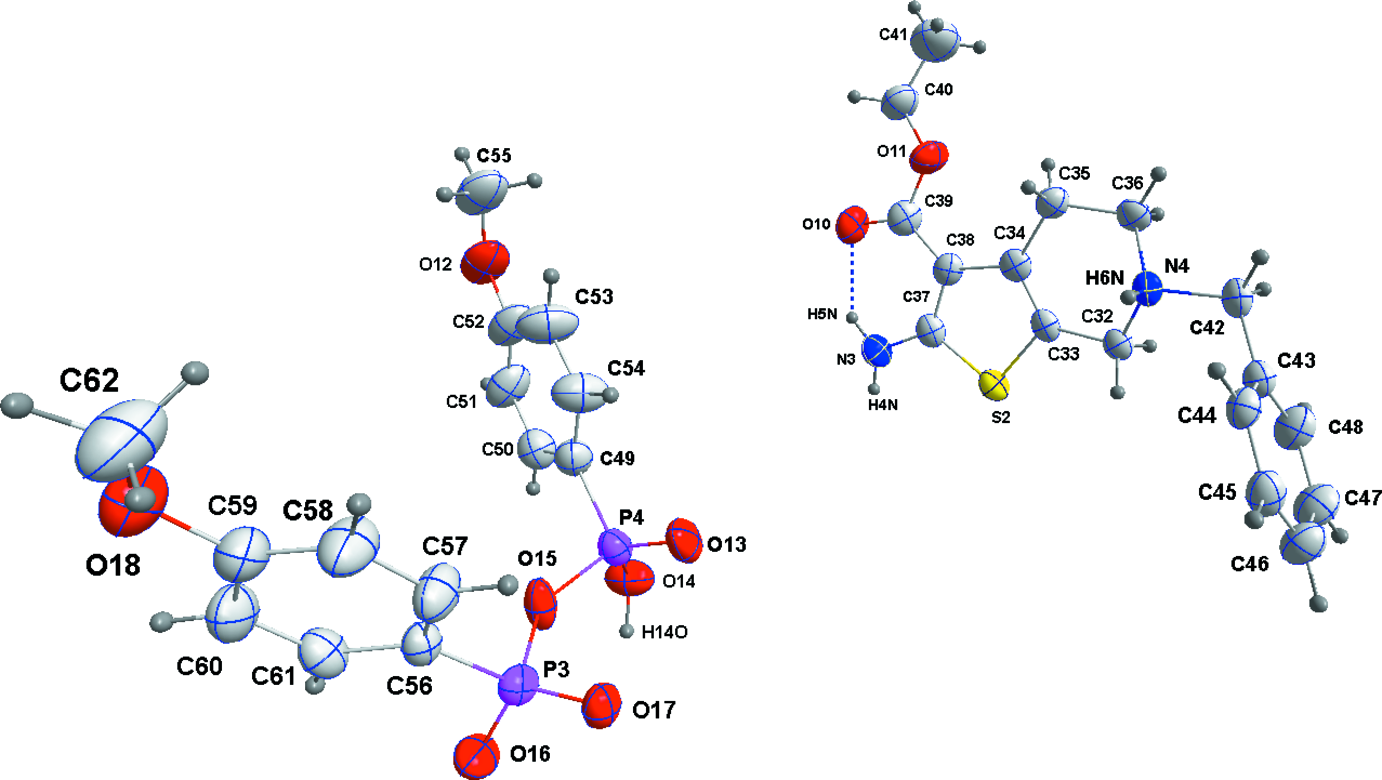

Supplement: Supplementary file 4 [file e-71-0o997-fig2.tif]

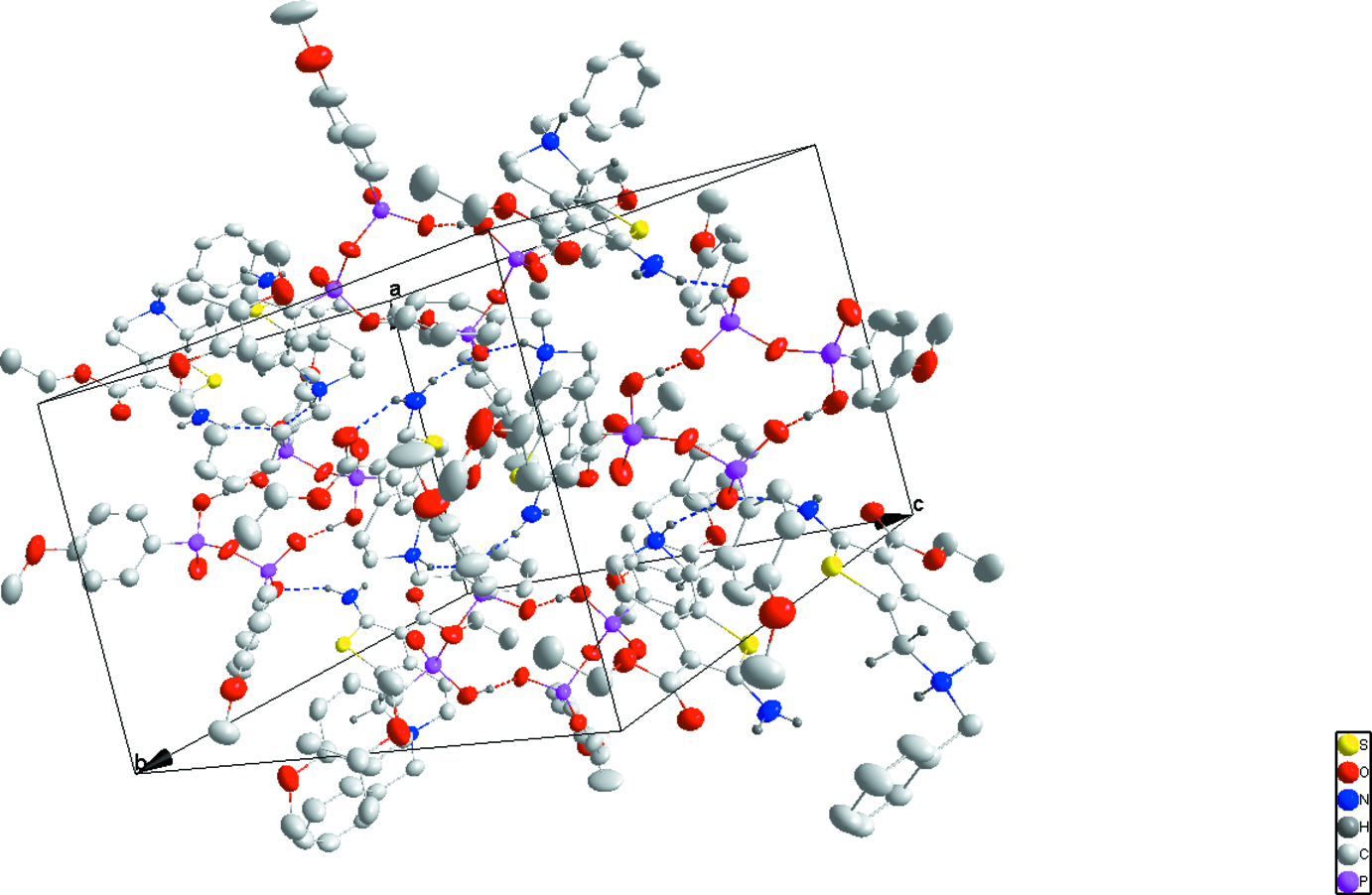

Supplement: Supplementary file 5 [file e-71-0o997-fig3.tif]
